# Supplementary material for: Preventive Health Behavior and Readiness for Self-Management in a Multilingual Adult Population: A Representative Study from Northern Italy
Source: Nurs Rep. 2025 Jul 1;15(7):240. doi: 10.3390/nursrep15070240 (PMC12299883; doi:10.3390/nursrep15070240)
Supplement: Supplementary file 1 [file nursrep-15-00240-s001.zip › nursrep-3676066-supplementary.pdf]

Supplementary Material

Table S1. Variance inflation factors (VIF) for all predictors included in the multivariable linear regression model of GHP-16 preventive health behavior scores (Table 3).

Variable	VIF
const	44.57
Female (vs. Male)	1.05
Age (in years)	1.31
Lives alone (vs. no)	1.04
Language (vs. German)	
Italian	1.12
Other	1.06
Education (vs. middle school or lower)	
Vocational school	1.79
High school	1.87
University	1.83
Works in health or social sector (vs. no)	1.10
HLS-EU-Q16 (vs. problematic) [1]	
Inadequate	2.36
Sufficient	2.76
Missing/unknown	2.17
PAM-10 (vs. disengaged and overwhelmed) [1]	
Becoming aware	2.14
Taking action	1.99
Maintaining	1.91

VIF values were calculated from the weighted linear regression model estimating associations between language group and preventive health behavior. All values were below the conventional threshold of concern (VIF < 5), indicating no evidence of multicollinearity among predictors. Reference categories are described in the Methods section.
